# Multilocular Disease of the Left Ovary

**Published:** 1874-05-15

**Authors:** A. Reeves Jackson

**Affiliations:** Surgeon-in-Chief of the Woman’s Hospital of the State of Illinois, etc.; 785 Michigan ave., Chicago


MEDICAL EXAMINER.

A

*Semi-Monthly Journal of Medical Sciences.*

EDITED BY N. S. DAVIS, M.D., AND F. H. DAVIS, M.D.

No. X. CHICAGO, MAY 15, 1874. VOL. XV.

### Original Communications.

### MULTILOCULAR DISEASE OF THE LEFT OVARY.—EXTENSIVE ADHESIONS.—OVARIOTOMY.—DEATH FROM SHOCK.

BY A. REEVES JACKSON, M.D., SURGEON-IN-CHIEF OF THE WOMAN'S HOSPITAL OF THE STATE OF ILLINOIS, ETC.

CATHERINE W., aged forty-seven years, unmarried, was admitted to the Woman's Hospital August 23d, 1873, and gave the following history:

She commenced menstruating at the age of fourteen, and the function was regularly and painlessly performed down to the age of thirty-eight, when it ceased to recur with regularity. During the following year, the flow appeared only at intervals of two and three months. At thirty-nine it ceased, and was absent for three years, during which time her health was excellent. Nearly five years ago she had an attack of bilious fever, which lasted four weeks. This was followed by a sanguineous discharge, which seemed to her like menstruation. She saw no return of it, however, until a year later, when a similar flow followed another attack of bilious fever. In the latter part of the year 1870, she had an illness which was pronounced congestion of the liver, during which she experienced pain along the edge of the ribs on the right side, extending over to the left, and also a pain which seemed to pass from the umbilicus through to the spine. At the same time, the abdomen increased in size, the enlargement being general, and not more in one part than another. This enlargement, and the pain in the hepatic region, lasted several weeks, and then together they passed slowly away. On the subsidence of the abdominal swelling, both her medical attendant and herself discovered a "cake-like"

tumor in the right ovarian region. It was "not round like an orange, but flattened, with uneven edges."

In February, 1872, she had another slight attack of fever, followed by several sanguineous discharges at irregular intervals, some of them lasting three or four days, and one of them a full week. Each of these hæmorrhages was preceded by pain, or rather a feeling of soreness, referred chiefly to the right ovarian region, and increased by certain movements of the body, as turning in bed, etc.

The tumor itself, although at times slightly tender under pressure, has never been the seat of pain.

Within the past eight months the abdomen has steadily and rapidly enlarged. In the latter part of last December she felt a good deal of soreness in the left side, followed by a sanguineous discharge, which was the last she has seen. Her health, latterly, has rapidly failed, and she has become very feeble. She has not been able to walk, both on account of debility and of a feeling of pressure about the left groin when she is on her feet. Her appetite, however, has been good; and her bowels have acted regularly.

*Present Condition.*—The patient is a tall woman, of large frame, but greatly emaciated. Her complexion is sallow; and her countenance presents the peculiar expression so frequently observed in persons with advanced disease of the ovaries, and which Mr. Spencer Wells has called the "*facies ovariana.*" The tongue is clean and smooth; pulse 120, small, feeble, and regular. The feet and ankles are œdematous. Moderate exertion causes rapid breathing. The abdomen is very much enlarged, measuring forty-five inches in circumference. The enlargement is not uniform, some portions being much more prominent than others. There is dullness on percussion everywhere except in a part of the right hypochondriac and right lumbar regions. Palpation shows the existence of an irregular tumor, extending from the brim of the pelvis to the ensiform cartilage, and passing under the edge of the left ribs, causing considerable bulging of the thorax on that side. On the right side it extends to a level with the edge of the false ribs. Over the right ovarion region there is a distinct roundish nodular projection, about the size of a cocoa-nut, which the patient identifies as the original tumor. To the left of it, and occupying a position near the median line of the body, are two smaller nodular projections. The great mass of the tumor, however, inclines to the left side. Fluctuation is evident, in varying degrees, in all parts of the tumor, and there is no tenderness in any part of it. The uterus is found to be normal as to position, size, and depth of cavity.

The diagnosis is multilocular disease of one or both ovaries.

The diagnosis having been subsequently confirmed by the other members of the hospital staff, and by Prof. J. W. Freer and Dr. Chas. G. Smith, of the Consulting Board, an operation was recommended as the only means of relief; and the patient, after being made fully aware of its dangerous character, and after consultation with her friends, decided to avail herself of this last resource.

*Operation.*—The patient having on the day previous been well purged by a full dose of castor-oil, and having eaten nothing but milk-porridge for twenty-four hours, the operation was performed Sept. 16th, 1873. There were present Drs. V. L. Hurlbut, W. C. Lyman, Flood, Emmons, and Blake, of the Hospital Staff, and, by invitation, Drs. C. G. Smith, McKennan, Harvey, and Chapman (of Hudson, Mich.); also, Messrs. Chapman, Harrington, and Moore, medical students.

The patient was placed upon the operating-table at 11 A.M., and being fully anæsthetized by Dr. Flood (alternately by chloroform and sulphuric ether), an incision, commencing an inch and a half below the umbilicus, was made in the linea alba, and extended directly downward about four inches. The different layers of the abdominal wall were successively divided until the peritoneum was reached. The bleeding from the incision having been checked by the application of sponges dipped in cold water, the abdominal cavity was cautiously opened with the point of a bistoury, and exit given to a small quantity of ascitic fluid. The small opening into the peritoneum was then enlarged to an inch and a half by means of scissors, a grooved director being previously used as a guide. The cyst-wall, having a pale bluish color, then appeared, filling up the opening. A silver catheter, having a large curve, was next introduced, and swept over the anterior and lateral walls of the tumor, for the purpose of ascertaining the position and extent of any existing adhesions. These were found to be very numerous and firm on both sides, more especially on the left. The abdominal incision was now extended downwards to five and a half inches. Bathing my hands in artificial serum, as first used and recommended by Dr. E. R. Peaslee,* I introduced the right and left successively, and separated all the parietal adhesions that could be reached. Many of these were very dense, and required much force for their rupture. The presenting portion of the cyst was then tapped with a large trocar, and about one pint of clear, amber-colored fluid escaped through the canula. In order to guard against the entrance of any of the cystic fluid into the peritoneal cavity, a piece of oiled silk was closely applied to the canula, and made to cover the lower portion of the incision. As the cyst collapsed, it was drawn through the opening, and other cysts were successively tapped by pushing the trocar through the canula, without withdrawing the latter. The fluid drawn from the different cysts varied greatly in color and consistence, as is usual in polycystic ovarian disease. The quantity of fluid removed in this manner did not exceed three or four pints; and its removal caused no sensible diminution in the size of the growth; I therefore removed the trocar, and, forcing the hand into the center of the tumor, broke down the intercystic septa in every direction. Even this procedure, however, failed to reduce its size sufficiently to enable me to draw it through the incision. This latter was, therefore, extended upward to the left of, and two

* Composed of chloride of sodium, four drachms; albumen (white of eggs), six drachms; water, four pints.

"It is intended to imitate the natural secretion of the peritoneum, and is kept at a blood-heat, and used to thoroughly moisten the operator's hands before they are introduced into the peritoneal cavity."—*Ovarian Tumors*, p. 402.

inches beyond, the umbilicus, and downward to within two inches of the symphysis pubis. The hand was then again introduced, and the surroundings of the tumor explored in every direction. Other adhesions were discovered and ruptured, and the tumor was then lifted from its bed and brought through the incision, this stage of the proceeding being greatly facilitated by turning the patient upon the left side. The tumor being held by assistants, the pedicle, consisting of the left broad ligament, was clamped as near the tumor as possible and divided. There were still several very strong bands of adhesion between the tumor and the pelvic walls. These were divided with scissors, and the tumor, being now wholly detached, was removed.

The right ovary was next examined, and found to be greatly atrophied, being no larger than a Lima bean. It was firm in texture, and whitish in color; it was undisturbed. The uterus was in all respects normal.

A good deal of blood was oozing from the torn and cut surfaces of the adhesions, and several minutes were consumed in checking it. The solution of the persulphate of iron was effectually used for this purpose in some parts, but in others it was found necessary to apply ligatures. The ends of these were, in all instances, cut off as closely as seemed consistent with safety.

The pedicle was next permanently treated. It was transfixed with a needle carrying a double ligature of well-waxed carbolized linen thread, tied in two parts, the ends of the ligatures cut off close, and the clamp removed.

As the patient began to exhibit signs of exhaustion, it was deemed prudent to hasten the remaining steps of the operation as much as possible. An india-rubber drainage-tube, one-fourth of an inch in diameter, perforated on alternate sides at intervals of half an inch, was passed from Douglas's cul-de-sac into the vagina. The end of the tube which was left in the abdomen was attached to a double silver wire, the free ends of which were twisted together and brought out through the lower angle of the incision. This step of the operation was accomplished as follows: A curved trocar and canula, guided by a finger, were passed into the posterior cul-de-sac of the vagina, and through the vaginal wall, the point of puncture and emergence being determined by pressure of a finger against the corresponding peritoneal surface immediately behind the uterus, a half inch from its vaginal attachment. The puncture having been made, the trocar was withdrawn. The lower end of the drainage-tube was then fastened to the abdominal end of the canula, and the latter withdrawn, carrying with it the tube through the vaginal canal.

The peritoneal cavity was then thoroughly cleansed, by means of carefully-prepared new sponges. This having been done, the wound was closed with thirteen silver-wire sutures, placed half an inch apart, each suture penetrating the entire thickness of the abdominal wall. A compress of folded flannel, wrung out of a warm, weak solution of carbolic acid, was laid over the incision, and over this a second larger compress, wet with hot water. A sufficient quantity of cotton-wool was placed over the compress, to give some rotundity to the now sunken abdomen, and this, in turn, was covered with a piece of oiled silk, the whole being finally secured in position by a flannel bandage passed around the body and pinned.

The time of the operation was one hour and forty minutes. Throughout the whole period the temperature of the room was kept at 76° to 80°, and the air was made moist by means of a large evaporating-dish filled with water and placed upon a stove.

The patient fully recovered from the influence of the anæsthetic; but so soon as she regained consciousness she complained of feeling tired. Her pulse was 120; respiration irregular and sighing. She took a half grain of sulphate of morphia, and had a tablespoonful of hot whisky-toddy every ten minutes. Hot blankets were laid over her, and india-rubber bags, filled with hot water, placed about her feet and legs. However, the pulse became more and more feeble, the breathing more irregular, and she expired in an hour and thirty minutes after the completion of the operation.

The weight of the tumor was thirty-five pounds.

*Remarks.*—An interesting fact in connection with the foregoing case was the discrepancy between its early history and the condition discovered at the time of operation. All the early symptoms pointed most unequivocally to the right ovary as the seat of disease, while the subsequent history developed the fact that they were really referable to the left.

Dr. W. L. Atlee* relates a case in which there was likewise a contradiction between the early symptoms and the subsequent physical signs. At the time of the operation, "*the tumor was most developed towards the left side*, and the percussion sound was *dull over the left lumbar* region, and elsewhere, except in the epigastric, right hypochondriac, and *right lumbar* regions, where it was *resonant*." These peculiarities pointed to the left ovary as the one affected; but "about three years before the patient had an attack of severe pain in the *right groin and hip*. One year after, her friends noticed an enlargement of which she was not aware. It extended uniformly over the whole lower portion of the abdomen. Afterwards, she had returns of pain in the right inguinal region." In this case, the diagnosis of disease of the right ovary, the correctness of which was confirmed by the operation, was based upon the early history; the distinguished author holding that when "the history of the case shows that in the early development of the tumor it had appeared in one or other groin, or that severe pain, in either side, had accompanied its origin, the side on which the early difficulty existed will determine which ovary is affected."

The case of Miss W. shows, however, that such a rule of diagnosis is not always reliable. Here, not only was the symptom of pain in the right side, but the early development of the tumor, also, so far as it was recognized; and yet, only the left ovary was enlarged.

785 Michigan ave., Chicago.

* *Ovarian Tumors*, p. 45.

